# 

**Published:** 1895-03

**Authors:** 


"WHEN WRITING TO ADVERTISERS, PLEASE SAY "I SAW YOUR AD IN THE ATLANTA MEDICAL AND SURGICAL JOURNAL."

ESTABLISHED 1855. SUBSCRIPTION, $2.00.

### ATLANTA MEDICAL AND SURGICAL JOURNAL.

LUTHER B. GRANDY, M.D.,
MANAGING EDITOR.

M. B. HUTCHINS, M.D.,

BUSINESS MANAGER.

COLLABORATORS:

H. V. M. MILLER, M.D., LL.D., VIRGIL O. HARDON, M.D., FLOYD W. McRAE, M.D., DUNBAR ROY, M.D., AND H. R. SLACK, M.D., PH.M.

VOLUME XII. NEW SERIES. ATLANTA, GA., MARCH, 1895. No. 1.

SURGEON GEN'L'S OFFICE 154157 LIBRARY.

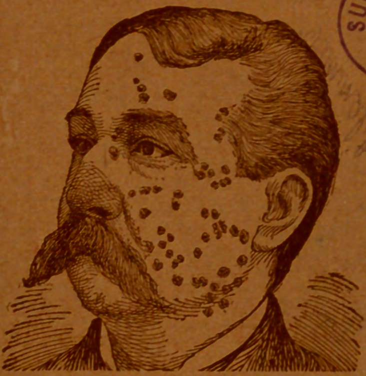

A STUBBORN CASE OF ACNE

MERCAURO
ARSENAURO

"Were administered alternately, beginning with 10 and increasing to 20 drops, 3 times daily—Redness and congestion disappeared at the end of fourth week. Skin cleared up at end of seventh week."

CHAS. ROOME PARMELE CO., 98 WILLIAM ST., N.Y.

PUBLISHED BY THE FRANKLIN PRINTING AND PUBLISHING CO., ATLANTA, GA.

Entered at the Post-Office at Atlanta, Ga., as second-class matter.

